# Supplementary material for: COVID-19 Vaccine Acceptance and Beliefs among Black and Hispanic Americans
Source: PLoS One. 2021 Aug 24;16(8):e0256122. doi: 10.1371/journal.pone.0256122 (PMC8384224; doi:10.1371/journal.pone.0256122)
Supplement: S1 File — (PDF) [file pone.0256122.s001.pdf]

COVID-19 Vaccine Survey

Thank you for agreeing to participate in this survey! Please answer the questions that follow. This survey is anonymous, so please be as open and honest as possible.

What is your age? (drop-down menu with ages in years)
(IF age<18, terminate survey and thank respondent)

Overall, how informed are you about COVID-19?

- Extremely
- Very
- Somewhat
- Not very
- Not at all

How worried are you about getting COVID-19?

- Extremely
- Very
- Somewhat
- Not very
- Not at all

Have you had or do you currently have COVID-19?

- Yes, I previously had COVID-19
- Yes, I currently have COVID-19
- No, I have never had COVID-19

(IF YES) How severe was your case of COVID-19?

- Extremely
- Very
- Somewhat
- Not very
- Not at all

(IF YES) Have you been hospitalized due to COVID-19?

- Yes
- No

Which of the following are true about COVID-19? Select all that apply.
COVID-19 is…

- Spread through the air.
- Created on purpose in a lab.
- Not that serious for most people.
- Being exaggerated.
- The worst public health crisis in decades.
- Being used to reduce personal freedoms.
- A hoax.
- No worse than seasonal flu.
- Spread through 5G.
- Spread by using products imported from other countries.
- Harder to catch if people wear masks.
- Cured by gargling disinfectants.
- A respiratory illness.
- More dangerous for people over age 60.
- None of the above.

Do you personally know anyone else who has been diagnosed with COVID-19?

- Yes
- No

(IF YES) How severe were their cases of COVID-19?

- Extremely
- Very
- Somewhat
- Not very
- Not at all

(IF YES) Were they hospitalized due to COVID-19?

- Yes
- No

How worried are you about other friends or family members getting COVID-19?

- Extremely
- Very
- Somewhat
- Not very
- Not at all

Which of the following describe you? Select all that apply.

- Healthcare worker.
- Skilled nursing or long-term care worker.
- 60 years old or older.
- Essential worker.
- First responder.
- Can work from home.
- None of the above.

As far as you know, are you in a priority group to get the COVID-19 vaccine?

- Yes
- No
- Not sure

How likely are you to get the COVID-19 vaccine when it becomes available?

- Definitely
- Probably
- Not sure
- Probably not
- Definitely not

Above you mentioned you would (PREVIOUS ANSWER) get the COVID-19 vaccine when it becomes available. Please explain, in detail, why you feel this way.
OPEN END

How often do you get the flu vaccine?

- At least once a year
- Every 2-3 years
- Every 4-5 years
- Rarely
- Never

How <u>informed</u> are you about the <u>COVID-19 vaccine</u>?

- Extremely
- Very
- Somewhat
- Slightly
- Not at all

What positive things, if any, have you heard about the COVID-19 vaccine?
OPEN END

What negative things, if any, have you heard about the COVID-19 vaccine?
OPEN END

What else have you heard about the COVID-19 vaccine?
OPEN END

After the COVID-19 vaccine is broadly available, how long do you plan to wait to get the vaccine?

- I want to get it immediately
- Up to 1 month
- 2-3 months
- 4-6 months
- 7-9 months
- 10-12 months
- More than one year
- I do not want to get the COVID-19 vaccine.

If a COVID-19 vaccine were available for free and in convenient locations, how likely would you be to get the vaccine?

- Definitely
- Probably
- Not sure
- Probably not
- Definitely not

If getting a COVID-19 vaccine required you to provide your name, address, or other identifying information, how likely would you be to get the vaccine?

- Definitely
- Probably
- Not sure
- Probably not
- Definitely not

How informed are you about <u>how the COVID-19 vaccine works</u>?

- Extremely
- Very
- Somewhat

- Not very
- Not at all

Which of the following are true about you? Select all that apply.
Thinking about how the COVID-19 vaccine works...

- I am not that interested.
- I don't know where to find information.
- Available information is too complex.
- I want to know more.
- I don't know enough about science to understand it.
- Please check this box.
- I know almost everything about the science behind it.
- None of the above.

Please read the following statements. Select the statements you agree with regarding the COVID-19 vaccine. Select all that apply.
Information about the COVID-19 vaccine is…

- Not available to me.
- Too technical.
- Presented in terms I am not familiar with.
- Overwhelming.
- Not trustworthy.
- Hard to understand.
- Just for scientists and doctors.
- Boring.
- Kept secret by drug companies.
- Kept secret by the government.
- Confusing.
- None of the above.

Please read the following statements. Select the statements you agree with regarding the COVID-19 vaccine. Select all that apply.

The COVID-19 vaccine is…

- Being rushed out too quickly.
- Dangerous.
- A miracle.
- More harmful than getting COVID-19.
- Going to cause people to catch COVID-19.
- Made from aborted fetuses.

- Going to be effective.
- Going to be distributed fairly.
- Not worth the risk.
- Going to have too many side effects.
- None of the above.

The COVID-19 vaccine is…
- Going to cause long-term health problems.
- Already being given to some people.
- Going to end the pandemic.
- Not going to be ready within the next 6 months.
- Just not something I trust.
- Going to be safe.
- Against God's will.
- Only really needed by elderly people and healthcare workers.
- Going to be available to rich people first.
- None of the above.

The COVID-19 vaccine is…
- An attempt by drug companies to make more money.
- Unnecessary.
- Poison.
- Going to be used to control people.
- Going to be used to track people's locations.
- An exciting scientific advancement.
- An example of how quickly society can solve big problems when people cooperate.
- Going to make people abandon masks and social distancing before it is safe to do so.
- Going to be used to alter people's DNA.
- None of the above.

Below are several more statements about the COVID-19 vaccine. Please select the statements you agree with. Select all that apply.

I am concerned that the COVID-19 vaccine is not being tested enough on...
- Children
- Teens
- Older people (60+ years old)
- African-Americans/ Blacks
- Hispanics/ Latinos
- Caucasians/ Whites

- Men
- Women who are not pregnant
- Pregnant women
- Other (specify)
- None of the above

Do you think COVID-19 will continue to surge after the vaccine is available?

- Yes
- Maybe
- No

Do your close friends and family members want you to get the vaccine?

- Yes
- Maybe
- No

Would you be likely to…

- Recommend to your friends/family that they get vaccinated.
- Recommend to your friends/family that they NOT get vaccinated.
- Not make a suggestion either way.

Please select all the statements below that describe you.
I generally trust information about the COVID-19 vaccine that comes from…

- The US government.
- Scientific research.
- My doctor or healthcare professional.
- Dr. Anthony Fauci.
- Donald Trump.
- Joe Biden.
- Oprah.
- Disney.
- Chris Hemsworth.
- Alex Rodriguez.
- LeBron James.
- Beyonce.
- Drug companies.
- Other (specify).
- None of the above.

I generally trust information about the COVID-19 vaccine that comes from…

- Taylor Swift.
- Barack Obama.
- Jennifer Lopez.
- Bill Gates.
- My religious leaders.
- My family members.
- My friends, coworkers or neighbors.
- My doctor or healthcare professional.
- Social media (e.g., Facebook, Twitter, Instagram).
- Television.
- Print newspapers or magazines.
- Please leave this box unchecked.
- Google or other search engines.
- Centers for Disease Control (CDC).
- World Health Organization (WHO).
- State Department of Health.
- Other (please explain).
- None of the above.

How much do you think medical professionals working on the COVID-19 vaccine are looking out for people of your race/ethnicity?

- Completely
- Mostly
- Somewhat
- Not much
- Not at all

(IF ABOVE < COMPLETELY) How can medical professionals working on the COVID-19 vaccine better look out for people of your race/ethnicity?

OPEN END

Would you feel more comfortable receiving the COVID-19 vaccine if the medical professionals working on it were of your same race/ethnicity?

- Yes
- Maybe
- No

Would you feel more comfortable receiving the COVID-19 vaccine if it were approved of and endorsed by people of your same race/ethnicity?

- Yes
- Maybe
- No

How <u>important</u> is it to you that <u>a medical professional of your race/ethnicity</u> endorses the vaccine before you take it?

- Extremely
- Very
- Somewhat
- Not very
- Not at all

What would make you feel <u>more comfortable</u> about receiving the COVID-19 vaccine? Select all that apply.

- More medical professionals of my race/ethnicity discussing it in the media
- More celebrities or social media influencers of your race/ethnicity discussing it in the media
- More information about side effects
- More testing of long-term effects
- More testing on people of my race/ethnicity
- More testing on people of my age
- More testing on people of my gender
- Other (specify)
- None of the above

Is your doctor or anyone else on your healthcare team of your same race/ethnicity?

- Yes
- Maybe
- No

Do you think the scientists developing the COVID-19 vaccine pay enough attention to how it affects people of your race/ethnicity?

- Yes

- Maybe
- No

How much do you think the government is looking out for the interests of people of your race/ethnicity?

- Completely
- Mostly
- Somewhat
- Not much
- Not at all

How strong is your sense of belonging to your racial/ethnic community?

- Extremely strong
- Very strong
- Somewhat very strong
- Not strong at all

The following questions are for classification purposes only.

Please select the statements that describe you. Select all that apply.

- I exercise three or more times per week.
- I use home remedies to cure illnesses.
- I get good healthcare.
- Please leave this box unchecked.
- I am a vegetarian.
- I use tobacco.
- I feel lonely.
- I feel anxious.
- I feel depressed.
- I consider myself religious.
- I wear a mask in public locations.
- I support the Black Lives Matter movement.
- I stay at home almost all the time to avoid COVID-19 exposure.
- I think COVID-19 guidelines are too restrictive.
- I think the recent presidential election was conducted fairly.
- None of the above.

(If DOES NOT check “I get good healthcare) Above, you indicated you do not get good healthcare. Which of the following are reasons for this?

- No health insurance.
- Too expensive.
- Lack of time.
- Work or family responsibilities.
- I don’t need healthcare because I know how to treat myself.
- Other (please specify).
- I actually do get good healthcare.

Below are some additional statements. Select all that apply.
In the past year, I have...

- Voted in an election.
- Experienced the death of a close family member or friend.
- Participated in a demonstration, protest, or political rally.
- Had trouble paying my rent or mortgage.
- Been laid off from a job.
- Bought a lottery ticket.
- Traded stocks or mutual funds.
- Had trouble paying bills.
- Had to cut back on household expenses.
- Saved money for retirement.
- Lost my health insurance.
- Purchased a new home or car.
- Been unable to find a job.
- Been unable to afford all the food I need.
- None of the above.

Which of the following have you used in the past 7 days? Select all that apply.

- Snapchat
- Parler
- Facebook
- Twitter
- Instagram
- Reddit
- TikTok
- Centers for Disease Control (CDC)
- Newsmax
- One America News (OAN)
- Fox News
- ABC News
- The Daily Show

- The New York Times
- CNN
- Colbert Report
- None of the above.

Which of the following activities have you done in the past 7 days? Select all that apply.

- Gone out to a restaurant, bar, club, or other place where people gather.
- Visited in person with older (60+ years) friends, relatives or neighbors.
- Gone to the grocery store or pharmacy.
- Gone to a friend, neighbor or relative's home (that is not your own).
- Had friends, neighbors or relatives over to your home.
- Gone to a family gathering where there were more than 10 people (e.g., reunion, funeral, birthday party).
- Gone to a gathering of friends where there were more than 10 people (e.g., party, wedding, or concert).
- Gone to a faith-based gathering where there were more than 10 people (e.g., church, synagogue, temple or mosque).
- Had groceries or food delivered to your home.
- None of the above

Which of the following health conditions do you have? Select all that apply.

- Asthma
- Obesity
- Depression
- Anxiety
- Lung disease
- Heart disease
- High blood pressure
- Diabetes
- Kidney disease
- Cancer within the past year
- Immunosuppressive condition
- None of the above.

Are you Hispanic or Latino?

- Yes
- No

What is your race? Select all that apply.

- White

- Black
- Asian/ Pacific Islander
- Native American/American Indian
- Other

What is your gender?

- Male
- Female
- Non-binary
- Other

What is the highest degree or level of school you have completed? *Select only one answer.*

- Less than high school
- Some high school
- High school graduate or equivalent (e.g., GED)
- Some college-no degree
- Associate’s degree (e.g., AA, AS) or technical degree
- Bachelor's degree (e.g., BA, BS, AB)
- Post Graduate degree (e.g., MD, PhD, MBA)

What is your relationship status? *Select only one answer.*

- Married
- Living with partner, but not married
- Widowed
- Divorced
- Separated
- Not married or living with partner

Which of the following best describes the area where you live?

- Urban
- Suburban
- Rural
- Other (specify)

Which of the following best describes your place of residence?

- Detached house
- Attached house or townhouse
- Apartment, flat
- Manufactured/mobile home

- Hotel or motel
- Rooming house or boarding house
- Boat or recreational vehicle
- Dormitory or group quarters
- Other (specify)

How many other people live with you at your place of residence?

- None- I live alone
- 1
- 2
- 3
- 4
- 5
- 6
- 7+

Of the (PREVIOUS ANSWER) people you live with, how many are children under age 18?

- 0
- 1
- 2
- 3
- 4
- 5
- 6
- 7+

In 2019, what was your total household income before taxes? Select only one answer.

- Less than $15,000
- $15,000 - $24,999
- $25,000 - $34,999
- $35,000 - $49,999
- $50,000 - $74,999
- $75,000 - $99,999
- $100,000 - $149,999
- $150,000 - $199,999
- $200,000 - $249,999
- $250,000 or more

In 2020, what was your total household income before taxes? Select only one answer.

- Less than $15,000
- $15,000 - $24,999
- $25,000 - $34,999
- $35,000 - $49,999
- $50,000 - $74,999
- $75,000 - $99,999
- $100,000 - $149,999
- $150,000 - $199,999
- $200,000 - $249,999
- $250,000 or more

Thank you for your help! You have completed the survey.
