# Supplementary material for: COVID-19 Vaccine Acceptance and Beliefs among Black and Hispanic Americans
Source: PLoS One. 2021 Aug 24;16(8):e0256122. doi: 10.1371/journal.pone.0256122 (PMC8384224; doi:10.1371/journal.pone.0256122)
Supplement: Attachment — Submitted filename: Response to Reviewer.docx [file pone.0256122.s005.docx]

Dear Dr. Alcendor:

Thank you so much for your thoughtful, insightful review of our manuscript, *COVID-19 Vaccine Acceptance and Beliefs among Black and Hispanic Americans*. Your commentary has helped us clarify our thinking and close gaps in this work. Furthermore, your review has raised questions for us that we believe will enrich our future research on the impact of race/ethnicity on health attitudes and outcomes. We are grateful for your generosity. Please see our responses to your comments below.

**1. Because the COVID-19 pandemic is changing daily, the authors should make a statement in the introduction that notes “at the time of this writing…….”**

References to evolving COVID-19 landscape have been added to the Introduction:

*The evolving COVID-19 pandemic…*

*At the time of this writing, approximately fifty thousand Black Americans and seventy thousand Hispanic Americans have died from the virus.*

**2. The available COVID-19 vaccines that have undergone Emergency Use Authorization should be included in the introduction because this information is changing daily and the updated information should be reflected in the manuscript. At the time of the survey performed for this study there were only 2 EUA approved vaccines, now there are 3, the J&J vaccine was approved February 27, 2021.**

We have made additions to the text, including in the Discussion:

*During the time this survey was conducted, the only widely known vaccines were the mRNA vaccines formulated by Pfizer-Biontech and Moderna. As additional vaccines become available, such as those from AstraZeneca and Johnson & Johnson, vaccine perceptions may change. Additional research may further analyze vaccine attitudes and beliefs as the pandemic evolves and new vaccines are available. In addition, as the pandemic continues, viral variants and mutations may impact attitudes towards vaccination. Further research should include questions assessing opinions towards vaccination in the context of viral variants, which may be more or less dangerous than the original SARS-CoV-2 virus.*

**3. The term “achieve broad-scale immunity” should be replaced by “herd immunity” which is define at this time to be between (75%-85%) of the US population.**

Changed as indicated, and added to the Introduction:

*Anderson et al. (2020) note that the vaccination levels required to achieve COVID-19 herd immunity may be up to 90%, making it necessary to achieve near-universal levels of vaccine acceptance.*

**4. The authors should mention the long-standing safety of vaccines given to children and adolescents to attend public school and the extremely low rate of adverse events in this population that represent 24% of the US population. Even more, we have to vaccinate enough of the global population to end this pandemic and we have to curtail the evolution of viral variants that could lead to this virus becoming endemic across the world not just in the US.**

We have made additions to the Introduction:

*Vaccine mandates, such as those required for school entry or public benefits, are common in many countries and have experienced wide public support, especially in the wake of disease outbreaks such as the 2015 measles outbreak in California and the 2017 measles outbreak in Italy (MacDonald et al., 2018). In their review, Spencer, Trondsen-Pawlowski and Thomas (2017) report the incidence of serious adverse advents from vaccinations is generally very small, especially when considered in light of the vaccines’ disease prevention benefits. Still, some studies have found that people of color may be more reluctant than Whites to vaccinate themselves and their children against diseases such as influenza. Black parents and patients have been shown to have higher concern about factors such as vaccine-related side effects (Santibanez et al., 2020). These concerns resulted in lower influenza vaccination rates: during the 2019-2020 flu season, the Centers for Disease Control (CDC) estimated that just 38% of Hispanic individuals and 41% of Black individuals received an influenza vaccine, compared to over half of non-Hispanic Whites. CDC data also indicates that Black and Hispanic Americans were especially likely to contract the flu, possibly due to vaccine reluctance compounded by the health impact of other social and economic inequities (Centers for Disease Control, 2020). The World Health Organization (WHO) has identified vaccine hesitancy as one of the top ten threats to global health, stating that vaccine hesitancy is a major factor driving the resurgence of measles (World Health Organization, 2019). Notably, the spread of misinformation, often through the media, may be a contributor to vaccine hesitancy in a variety of groups (Nagler et al., 2020; Dubé et al, 2016; Habib et al., 2021).*

*In the era of COVID-19, vaccine hesitancy may have even greater consequences. Novel and dangerous SARS-CoV-2 variants continue to emerge, making the pursuit of herd immunity through wide-scale, global vaccination an urgent imperative. Breaking the chain of disease transmission for SARS-CoV-2 is critical. Anderson et al. (2018) discussed the impact of child vaccination programs and observed that beyond the direct effect on the children who are vaccinated, child vaccination also provides substantial indirect community protection. By blocking transmission, vaccinated people of all ages prevent exposure to unvaccinated members of their communities. Anderson et al. (2018) further note that when new childhood vaccines are implemented, disease incidence significantly declines throughout the entire population, even among those who are unable to receive the vaccines themselves.*

**5. There is also not one sentence about viral variants and their contribution to the pandemic.**

We have corrected this in response 4 above, as well as in the Discussion:

*In addition, as the pandemic continues, new viral variants and mutations may impact attitudes towards vaccination. Further research should include questions assessing opinions towards vaccination in the context of viral variants, which may be more or less dangerous than the original SARS-CoV-2 virus.*

**6. Vaccine hesitancy among healthcare providers should be mentioned in the manuscript.**

We have added the following to the Discussion:

*Given this, healthcare providers’ own vaccine status and attitudes are particularly important so that they can accurately inform their patients. However, healthcare worker COVID-19 vaccine hesitancy itself is a potential challenge. Lucia, Kelekar and Afonso (2020) found that nearly a quarter of medical students were reluctant to receive the vaccine upon regulatory approval (also see Kukreti et al., 2021 and Saied et al., 2021). Paterson et al. (2016) note that making vaccination for healthcare providers convenient and inexpensive, as well as increased training about vaccines and patient communication strategies, can increase vaccine compliance among patients. Given the influential role that Black and Hispanic medical professionals play in encouraging vaccine uptake among Black and Hispanic patients, it is critically important to make it as easy as possible for them to receive the vaccine. US Blacks and Hispanics in this study were significantly more likely than Whites to state that more testing of the vaccine on their racial/ethnic group is needed. Researchers, too, have noted the challenge of achieving adequate representation of Black and Hispanic patients in medical research (e.g., Fayanju et al., 2019; Cunningham-Erves, 2020). Our results suggest that the effectiveness and safety of the vaccines among minority study participants should be further highlighted. Promoting the dissemination of accurate research information, including the experiences of minority vaccine recipients, could demonstrate the positive effects of the vaccine in these groups, encouraging them to see the vaccine as safe and effective and increasing vaccination uptake.*

**7. There is no information about participants other than age, race, gender, and ethnicity. There is no information on their socioeconomic status, if they have access to the internet, low vs high income, education level, political affiliation, and do they have insurance etc. There many factors that influence attitudes about this vaccine.**

We have added an additional table summarizing demographics (Table 1) to the Results.

Furthermore, your comments helped us better articulate our perspective on when statistical controls are appropriate, especially when considering the effects of variables that are not just correlated and thus can be validly partialled out through analytic techniques, but are so causally enmeshed they are difficult to meaningfully separate. In the Discussion, we have added this as a direction for further research:

*Given the observed racial/ethnic differences in median income, it is reasonable to ask whether race/ethnicity effects observed may actually be driven by this confounding factor. However, race is an inherently social construct. In present day United States, one social component of being Black or Hispanic is a high likelihood of experiencing systemic racism in everyday life. Systemic racism acts through a pervasive variety of mechanisms, such as exclusion and bias, to significantly reduce access to valued cultural assets like education and income. In other words, education and income can have both direct effects on COVID-19 vaccine attitudes, as well as serving as mediating variables between race/ethnicity and COVID-19 vaccine attitudes (see Baron and Kenny, 1986). Data modeling from a limited sample, observational study has inadequate ability to account for mediating relationships of this type. In order to control for such a variable, other methods, such as experimentation or longitudinal measurement, should be explored in future research.*

**8. The other category in Table 1 should be briefly defined.**

We have added the following to the Methodology section:

*Analysis focused primarily on the results from the largest three of these five groups: White, Black and Hispanic, given the small sample size for the Asian group and the difficulty in meaningfully interpreting results from the Other category, which was comprised of a variety of different racial and ethnic groups, such as such as American Indian, Middle Eastern and multiracial individuals.*

**9. Relating these findings to participant education, income levels, political affiliation etc could have profound effects on the data.**

We agree, and intend to conduct future research to ask a broader array of demographic questions among a wider sample in order to develop causal models in this area. We have added Table 1 to the paper to increase the readers’ access to these data points, to the extent they are available from our research.

**10. The participants location in the US (rural vs suburban, South vs West) could also be mentioned and would likely have a profound effects on the results of this study.**

We have added Table 1 to the paper to provide more information about the demographic characteristics of the respondents, including Rural/Urban/Suburban distinctions.

**11. African Americans and Hispanic-Latin-x communities are culturally distinct and will require a culturally competent approach which should be mention as a part increasing vaccine acceptance and reducing vaccine hesitancy in minority communities.**

We have added the following to the Discussion:

*As the Black American and Hispanic American communities are culturally distinct and each contain a variety of ethnic groups, it is important to develop comprehensive communications that take the diversity of these communities into account. Culturally competent approaches are necessary when developing the most successful methods to increase vaccine acceptance among minority groups.*

Thank you again for your consideration and efforts in helping us improve this work.

Best regards,

Katherine Kricorian
