# Supplementary material for: COVID-19 Vaccine Acceptance and Beliefs among Black and Hispanic Americans
Source: PLoS One. 2021 Aug 24;16(8):e0256122. doi: 10.1371/journal.pone.0256122 (PMC8384224; doi:10.1371/journal.pone.0256122)
Supplement: Attachment — Submitted filename: Alcendor review PONE-D-21-04327 - R1 reviewer R1.doc [file pone.0256122.s006.doc]

June 12, 2021

Editor and Chief
PLOS ONE

Manuscript ID: PONE-D-21-04327

Dear Editor,

My comments regarding manuscript PONE-D-21-04327R1 entitled **“COVID-19 Vaccine Acceptance and Beliefs among Black and Hispanic Americans”** are enclosed.

Thank you for giving me another opportunity to review this revised manuscript submitted to your journal for publication.

Kind regards,

Donald J. Alcendor, Ph.D.

Associate Professor

Meharry Medical College

Center for AIDS Health Disparities Research

& Department of Microbiology and Immunology

& Obstetrics and Gynecology

Hubbard Hospital 5th Floor Rm. 5025

1005 Dr. D.B. Todd Jr. Blvd.

Nashville, TN 37208

Phone: 615-327-6449

Fax: 615-327-6929

Email: dalcendor@mmc.edu

Associate Professor Adjunct

Department of Pathology, Microbiology and Immunology

Vanderbilt University Medical Center

**REVIEW**

Dear Author,

The manuscript examines COVID-19 vaccine acceptance and beliefs among Black and Hispanic Americans. The paper is part of a broad, representative survey of US adults (N=1950) in order to better understand vaccine beliefs and explore opportunities to increase vaccine acceptance among these groups. The survey results suggested that Black and Hispanic individuals were less willing than Whites to receive the COVID-19 vaccine immediately.

**Overall:** The manuscript is fairly well written and my comments have been adequately addressed in the revised manuscript. It is my opinion that the manuscript should now be considered for publication.

**Main Comments:**

1. Because the COVID-19 pandemic is changing daily, the authors should make a statement in the introduction that notes “at the time of this writing…….”

*“At the time of this writing, approximately fifty thousand Black Americans and seventy thousand Hispanic Americans have died from the virus.” A reference for this statement would have been nice.* This comment has been addressed in the revised manuscript.

2. The available COVID-19 vaccines that have undergone Emergency Use Authorization should be included in the introduction because this information is changing daily and the updated information should be reflected in the manuscript. At the time of the survey performed for this study there were only 2 EUA approved vaccines, now there are 3, the J&J vaccine was approved February 27, 2021.

This comment has been addressed in the revised manuscript.

3. The term “achieve broad-scale immunity” should be replaced by “herd immunity” which is define at this time to be between (75%-85%) of the US population.

This comment has been addressed in the revised manuscript.

4. The authors should mention the long-standing safety of vaccines given to children and adolescents to attend public school and the extremely low rate of adverse events in this population that represent 24% of the US population. Even more, we have to vaccinate enough of the global population to end this pandemic and we have to curtail the evolution of viral variants that could lead to this virus becoming endemic across the world not just in the US.

This comment has been addressed in the revised manuscript.

5. There is also not one sentence about viral variants and their contribution to the pandemic.

This comment has been addressed in the revised manuscript.

6. Vaccine hesitancy among healthcare providers should be mentioned in the manuscript.

This comment has been addressed in the revised manuscript.

7. There is no information about participants other than age, race, gender, and ethnicity. There is no information on their socioeconomic status, if they have access to the internet, low vs high income, education level, political affiliation, and do they have insurance etc. There many factors that influence attitudes about this vaccine.

This comment has been addressed in the revised manuscript.

8. The other category in Table 1 should be briefly defined.

This comment has been addressed in the revised manuscript.

9. Relating these findings to participant education, income levels, political affiliation etc could have profound effects on the data.

This comment has been addressed in the revised manuscript.

10. The participants location in the US (rural vs suburban, South vs West) could also be mentioned and would likely have a profound effect on the results of this study.

This comment has been addressed in the revised manuscript.

11. African Americans and Hispanic-Latin-x communities are culturally distinct and will require a culturally competent approach which should be mention as a part increasing vaccine acceptance and reducing vaccine hesitancy in minority communities.

This comment has been addressed in the revised manuscript.
