# Supplementary material for: COVID-19 Vaccine Acceptance and Beliefs among Black and Hispanic Americans
Source: PLoS One. 2021 Aug 24;16(8):e0256122. doi: 10.1371/journal.pone.0256122 (PMC8384224; doi:10.1371/journal.pone.0256122)
Supplement: Attachment — Submitted filename: Responses to reviewer COVID-19 Vaccine Hesitancy 07.07.2021.docx [file pone.0256122.s007.docx]

Dear Reviewer:

Thank you for your thorough review of our manuscript, COVID-19 Vaccine Acceptance and Beliefs among Black and Hispanic Americans. We appreciate your thoughtful comments and are grateful for the opportunity to sharpen our work based on your feedback. Please see our responses to your comments below.

***Primary Concerns that should be addressed:***

***My main concern is that H4 essentially arises from nowhere, with the background of the paper focusing exclusively on why we would expect racial/ethnic differences, and provides essentially no motivation for this hypothesis, or even why this particular question is an additional focus of the paper. There is ample existing research to draw on to provide support for this hypothesis, but as written this is missing from the current manuscript.***

We have deleted H4 as a hypothesis and now report the results from those survey questions as exploratory findings. Our intent in including these questions in the survey is to provide potential directions for developing communication plans for addressing the other hypothesized findings.

***In the text, the authors suggest that care was taken to compare the sample to US composition, and re-weight to adjust for that representation, but no details are provided for those comparisons (e.g., in Table 1) or weighting approaches.***

We have added the following on page 5 to provide more detail on the data weighting:

Population values were developed using data from the 2019 US Census American Community Survey. Population percentages were calculated for the matrixed percentage values for age range, gender, race/ethnicity, income range and Census region, and were compared to the same matrixed percentage values within the survey sample. Survey responses within each Age range * Gender * Race/ethnicity * income range * Census region cell were upweighted or downweighted so that the resulting percentages, shown in table 1, reflected the population distribution present in the ACS results. Most cell weights differed negligibly from 1.0.

**Lesser Concerns for the authors to consider:**

***A previous reviewer asked how the observed associations are patterned by indicators of SES. The authors claim that such multivariate analyses are not feasible with these data. While the structure may not allow for formal mediation analyses, they seem more than sufficient to include one (or more) of these as controls in a multivariate model. That is, the paragraph on p. 19 is an unconvincing response to this previous reviewer request. I’m not convinced this paper needs that additional layer; but it’s not infeasible as the authors suggest.***

We have revised this section on page 21 of the tracked changes manuscript as follows:

Ideally, the data analysis would have controlled for the level of income and/or education of survey respondents in order to clarify effect drivers. Given the observed racial/ethnic differences in median income, it is reasonable to ask whether any race/ethnicity effects observed in the analysis may actually be driven by these confounding factors. Further examination of these effects is an opportunity for future research.

***The phrase “end the pandemic” (p. 1) is not especially meaningful. The likely outcome is not that SARS-COV-2 will fade away, but that it will become endemic in ways that are manageable at a population level. In other words, this common phrase often brings to mind an unlikely eventuality, and isn’t very helpful here.***

We have deleted this phrase.

***The range of estimates are wide for herd immunity. It’s unclear why the authors draw only on high-end estimates, suggesting the need for near universal vaccine coverage. If anything, this focus is likely to undermine the seeming aim of the paper to increase vaccine uptake.***

We have added the following on page 2 of the tracked changes manuscript:

However, Dong et al. hypothesized that vaccination of 60% of the population may be sufficient. Randolph and Barriero noted that herd immunity thresholds can vary widely within populations and are non-uniformly distributed, implying that a single standard for herd immunity may be an oversimplification.

***The responses to the timing question (Table 3) could be substantially affected by perceived timing of availability / access to the vaccine. In other words I worry that responses here could essentially be double barreled in that they may capture both how long someone perceives it will be before the vaccines is available to them, and how long they would wait after that point, while it’s only being interpreted to represent the latter. Is there any reason to not be concerned about this? If not, a caveat should probably be made about its interpretation.***

The specific wording of the survey question was “After the COVID-19 vaccine is broadly available, how long do you plan to wait to get the vaccine?” However, the Table 3 heading implies that the survey question wording was the potentially double barreled “How long do you plan to wait to get the COVID-19 vaccine?” We have corrected the table heading and text references on page 10 to clarify this more nuanced question wording.

***The text on p. 12 and the table on p. 13 don’t match. The text suggests the comparisons being made include those reporting both extremely and very important, while it’s clear they only include the “extremely” row from the table.***

We have corrected this.

***- “Data” are plural. So “CDC data also indicate” (not “indicates”) on p. 2.***

***- Methodology is the study of methods. A study’s design and analytic strategies are its “Methods” (p. 4).***

***- “…respondents were less likely to be…” (not “like) on p. 6.***

We have corrected these errors.

Thank you again for your thoughtful review of this manuscript and for providing such constructive feedback. We appreciate your help.

Best regards,

Katherine Kricorian
